# A Comparative Evaluation of Tools to Predict Metabolite Profiles From Microbiome Sequencing Data

**DOI:** 10.3389/fmicb.2020.595910

**Published:** 2020-12-04

**Authors:** Xiaochen Yin, Tomer Altman, Erica Rutherford, Kiana A. West, Yonggan Wu, Jinlyung Choi, Paul L. Beck, Gilaad G. Kaplan, Karim Dabbagh, Todd Z. DeSantis, Shoko Iwai

**Affiliations:** ^1^Second Genome Inc., Brisbane, CA, United States; ^2^Altman Analytics LLC, San Francisco, CA, United States; ^3^Department of Medicine, University of Calgary, Calgary, AB, Canada

**Keywords:** metabolome, human microbiome, computational prediction, metabolic potential, Next Generation Sequence

## Abstract

Metabolomic analyses of human gut microbiome samples can unveil the metabolic potential of host tissues and the numerous microorganisms they support, concurrently. As such, metabolomic information bears immense potential to improve disease diagnosis and therapeutic drug discovery. Unfortunately, as cohort sizes increase, comprehensive metabolomic profiling becomes costly and logistically difficult to perform at a large scale. To address these difficulties, we tested the feasibility of predicting the metabolites of a microbial community based solely on microbiome sequencing data. Paired microbiome sequencing (16S rRNA gene amplicons, shotgun metagenomics, and metatranscriptomics) and metabolome (mass spectrometry and nuclear magnetic resonance spectroscopy) datasets were collected from six independent studies spanning multiple diseases. We used these datasets to evaluate two reference-based gene-to-metabolite prediction pipelines and a machine-learning (ML) based metabolic profile prediction approach. With the pre-trained model on over 900 microbiome-metabolome paired samples, the ML approach yielded the most accurate predictions (i.e., highest F1 scores) of metabolite occurrences in the human gut and outperformed reference-based pipelines in predicting differential metabolites between case and control subjects. Our findings demonstrate the possibility of predicting metabolites from microbiome sequencing data, while highlighting certain limitations in detecting differential metabolites, and provide a framework to evaluate metabolite prediction pipelines, which will ultimately facilitate future investigations on microbial metabolites and human health.

## Introduction

The scientific community has only recently begun to realize and fully appreciate the significant role of the microbiome in human health (Turnbaugh et al., 2007; Integrative HMP (iHMP) Research Network Consortium, 2014). Increased access to high-throughput sequencing technologies has facilitated a record number of metagenomic- and metatranscriptomic-based investigations of host tissues and the microbial communities they support, which have begun to shed light on the pivotal impact of this ecosystem’s eubiosis in human health. Omics technologies empower mechanistic and therapeutic discovery relating to disease onset, progression, and treatment (Metwaly and Haller, 2019; Zhang et al., 2019). Small molecules are key factors in all host-microbe interactions, which can be synthesized, metabolized, and even modified by specific microbial taxa. The downstream effects of such metabolite modulation have been implicated in biological processes germane to human health (Zierer et al., 2018; Descamps et al., 2019). For example, researchers have shown that the microbial metabolite trimethylamine-N-oxide (TMAO) is a predictive marker of cardiometabolic diseases (Ussher et al., 2013; Ufnal et al., 2015; Li et al., 2018), as have secondary bile acid metabolites on immune system homeostasis and glucose and lipid metabolism (Molinero et al., 2019; Song et al., 2019), and microbial-derived gamma-aminobutyric acid (GABA) as a neurotransmitter of the central nervous system (Bravo et al., 2011; Strandwitz et al., 2019; Zheng et al., 2019).

Untargeted metabolomic analyses greatly facilitate the detection and characterization of a wide range of metabolites, affording researchers a comprehensive understanding of the metabolic pathways invoked within a microbial community. Such techniques have bolstered and accelerated mechanistic studies and biomarker identification strategies across a variety of diseases (Li et al., 2016; Franzosa et al., 2019; Glinton and Elsea, 2019; Urpi-Sarda et al., 2019). With massively large amounts of raw microbiome sequencing data being deposited into public sequence repositories at ever-increasing rates, we hypothesized that it is possible to predict metabolic profiles based solely on the sequencing data from a microbial community. After all, an accurate and reliable *in silico* means of predicting metabolic capacity from nucleic acid sequences would embolden drug discovery by generating testable hypotheses *sans* cost-prohibitive upstream metabolome profiling analyses.

Recent years have seen advances in linking microbiome sequencing data to metabolome data. One such strategy relies on the network of connections linking a given gene to reactions and compounds in a database. These linkages are used to infer molecular compound identities from the genetic information housed within a microbial community. A method called predicted relative metabolomic turnover (PRMT) was used to predict metabolites from a coastal marine metagenomics dataset, and the predicted metabolites correlated strongly with environmental factors (Larsen et al., 2011). MIMOSA was later developed to predict metabolic potential in a given microbial community and identify the microbial taxa most responsible for the synthesis/consumption of key metabolites (Noecker et al., 2016). Capitalizing on plentiful abundances of gene-to-metabolite data housed in repositories like KEGG, these utilities promote the generation of testable hypotheses and identification of potential drug targets (Chang et al., 2019). MIMOSA has been successfully applied in a number of studies to identify the microbial origin of certain metabolites (Stewart et al., 2018; Sharon et al., 2019). Meanwhile, interested in metabolites that directly associate with genes regardless of the reaction network and not limited to the KEGG database, we developed Mangosteen: a metabolome prediction pipeline dependent upon relationships between KEGG/BioCyc reactions and the molecular compounds directly associated with those reactions. Both MIMOSA and Mangosteen are reference-based, and as such, they rely heavily on the completeness and accuracy of the database queried. As the vast majority of microbial taxa belonging to the human gut microbiome remain unknown (Turnbaugh et al., 2007; Sunagawa et al., 2013), predictions from these reference databases provide a partial view of the metabolic capacity housed within a community.

Mallick et al. (2019) devised MelonnPan, which exploits machine learning (ML) to predict metabolomic potential. *Via* MelonnPan, a model trained from paired microbiome and metabolome datasets can be used to predict metabolites from a novel microbiome dataset *san a priori* knowledge regarding relationships between genes and metabolites. This approach circumvents the limitations of the reference-based methods discussed above and has been used to generate promising results between two inflammatory bowel disease (IBD) cohorts (Franzosa et al., 2019; Mallick et al., 2019).

**FIGURE 1:**
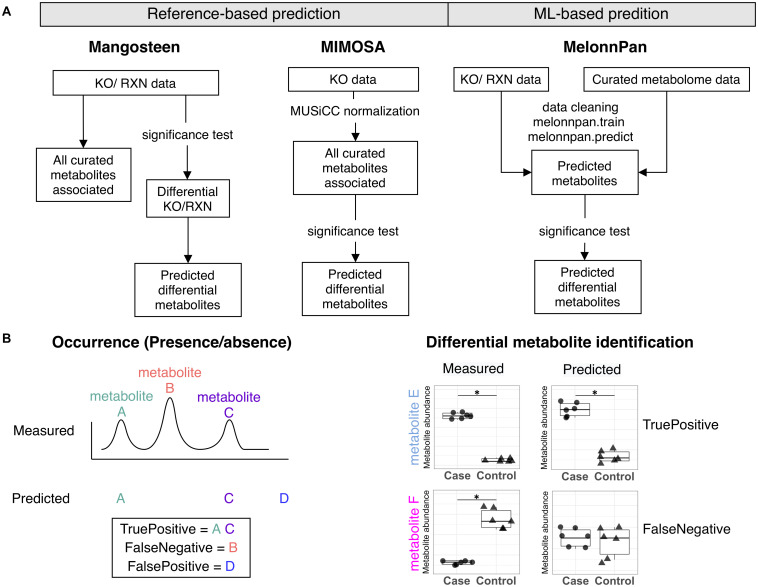
**(A)** Metabolite prediction workflow for Mangsoteen, MIMOSA, and MelonnPan. **(B)** Evaluation metrics used to appraise prediction performance regarding occurrence and differential metabolite identification.

Despite the development and refinement of these pioneering pipelines, to date there has not been a thorough comparison of reference-based and ML-based techniques. Ergo, we comparatively analyzed the performance of two reference-based methods, Mangosteen and the compound prediction component of MIMOSA, and the ML-based MelonnPan approach in conducting microbiome metabolite predictions (Figure 1). A detailed evaluation was performed on occurrence, abundance, and between-group differences against metabolome data acquired *via* empirical measurements (Figure 1B).

## Materials and Methods

### Data Collection

A PubMed search querying the keywords “gut microbiota” OR “gut microbiome” OR “fecal microbiota” OR “fecal microbiome” AND “metabolome” AND “humans” resulted in 171 papers (Supplementary Figure 1). Filtering was applied to remove studies that (1) were not original research articles, (2) were conducted *in vitro* or in newborn and/or infant subjects, (3) consisted of samples other than stool or intestinal tissue, (4) had microbiome data other than NGS sequencing, (5) had targeted metabolomics data, and (6) had fewer than 30 microbiome-metabolome paired samples. Notably, as different metabolome generation and processing methods have significant impact on results, we only selected studies which applied untargeted metabolome profiling with multiple liquid chromatography-mass spectrometry (LC-MS) methods or nuclear magnetic resonance (NMR) for a comprehensive view of the metabolite pool. Eighteen studies were retained and six were used in this evaluation due to data accessibility (Table 1).

**TABLE 1:** Characteristics of datasets included for prediction and evaluation.

Disease area – sample type	Study	Treatment group (counts of biospecimens in each group)	Metabolome dataset	Microbiome dataset			Contrast for differential analysis
				
			Profiling technology	Target region	Profiling technology	Data availability	
Healthy – Stool samples	Maier et al., 2017	High resistant starch intervention (14) Low resistant starch intervention (14) Baseline control (13)	SolariX Fourier transform ion cyclotron resonance mass spectrometer (FT-ICR-MS; Bruker Daltonik GmbH)	16S rRNA gene: V4-V6	HiSeq 2000 (Illumina)	EBI-ENA accession: ERP104494	High resistant starch intervention: Baseline control
Colorectal cancer (CRC) – Biopsy samples	Hale et al., 2018	Diseased tissue (28) Healthy tissue distal to diseased tissue (35) Healthy tissue proximal to diseases tissue (20)	Quadruple time-of-flight mass spectrometer (Agilent Technologies 6550 Q-TOF)	16S rRNA gene: V3-V5	MiSeq (Illumina)	NCBI SRA BioProject: PRJNA445346	Diseased tissue: Healthy tissue distal to diseased tissue
Autism spectrum disorder (ASD) – Stool samples	Kang et al., 2018	ASD (23) Healthy control (21)	Varian Direct Drive (VNMRS) 600 MHz spectrometer (Agilent Technologies)	16S rRNA gene: V2-V3	Genome Sequencer FLX-Titanium System (Roche)	Qitta: study ID 11169	ASD: Healthy control
Inflammatory bowel disease (IBD) – Stool samples	Franzosa et al., 2019	Crohn’s disease (88) Ulcerative colitis (76) Non-IBD Control (56)	Q Exactive Hybrid Quadrupole-Orbitrap mass spectrometer; Exactive Plus Orbitrap mass spectrometer (Thermo Fisher Scientific)	Whole genome	HiSeq 2500 (Illumina)	NCBI SRA BioProject: PRJNA400072	Crohn’s disease: Non-IBD Control
	Lloyd-Price et al., 2019	Crohn’s disease (139) Non-IBD Control (86)	Q Exactive/Exactive Plus orbitrap mass spectrometers (Thermo Fisher Scientific)	Whole genome	HiSeq2000; HiSeq 2500 (Illumina)	NCBI SRA BioProject: PRJNA398089	Crohn’s disease: Non-IBD Control
				Whole transcriptome	HiSeq2500 (Illumina)		
	SG_IBD (generated in this study)	Active ulcerative colitis (10) Inactive ulcerative colitis (19) Healthy control (15)	Q Exactive orbitrap mass spectrometers (Thermo Fisher Scientific)	16S rRNA gene: V4	MiSeq (Illumina)	NCBI SRA BioProject: PRJNA668188	Active ulcerative colitis: Healthy control
				Whole transcriptome	NextSeq 550 (Illumina)		

### Microbiome Sequencing Data Pre-processing

Raw sequencing data were either downloaded from NCBI Sequence Read Archive (SRA) for public studies or generated in-house using the Illumina platform (Table 1). We analyzed 16S rRNA amplicon Illumina sequencing data using DADA2 (Callahan et al., 2016) and subjected to functional composition prediction *via* Piphillin with identity set at 0.97 (Iwai et al., 2016; Narayan et al., 2020). For 16S rRNA amplicon pyrosequencing data, we compared it to StrainSelect (strainselect.secondgenome.com) using USEARCH (Edgar, 2010). We assigned a strain operational taxonomic unit (OTU) to sequences matching a unique strain with a global alignment identity ≥99% and with the highest identity to a single strain. To ensure specificity of these strain matches, a difference ≥0.25% between the identity of the best and second-best match was required (e.g., 99.75 vs. 99.5). All remaining non-strain sequences were quality filtered and dereplicated with USEARCH. We then clustered the resulting unique sequences at 97% using UPARSE (*de novo* OTU clustering) and determined a representative consensus sequence per *de novo* OTU. Piphillin (Iwai et al., 2016; Narayan et al., 2020) was then applied to the OTU table to infer community function.

For metagenomic and metatranscriptomics reads, adapter sequences and low-quality ends were trimmed with Trimmomatic (<Q20; Bolger et al., 2014). We then removed contaminant sequences, e.g., PhiX174 and sequencing primers, using Bowtie2 (Langmead and Salzberg, 2012). For metatranscriptomic data, all rRNA sequences from all three domains of life were identified and removed from consideration using SortMeRNA 2.0 (Kopylova et al., 2012). Host sequences were omitted using Kraken (Wood and Salzberg, 2014), which used exact matches of raw shotgun sequences to k-mers derived from the human reference genome. Filtered DNA sequences were mapped against a custom database built from KEGG (May 2019 release; Kanehisa et al., 2011) and BioCyc (version 23.0; Karp et al., 2017). Specifically, we collected protein sequences of bacteria, fungi and viruses from KEGG as well as protein sequences in MetaCyc and all PGDBs in BioCyc followed by a de-replication step at 0.99 identity and 0.99 alignment coverage using CD-HIT (Li and Godzik, 2006; Fu et al., 2012). A search for translated DNA sequences was executed using Diamond (Buchfink et al., 2014) and hits that spanned ≥ 20 amino acids with ≥80% similarity were retained. Upon identifying multiple hits, reads were equally split between the best hits.

### Metabolite Identifier Curation

Detailed LC-MS/NMR conditions, software and library databases for metabolite data generation per each study are listed in Supplementary Table 1. These annotated metabolites were assigned unique identifiers corresponding to an in-house chemical dictionary so as to facilitate comparison between studies. KEGG, BioCyc, the PubChem Identifier Exchange Service^1^, and the Chemical Translation Service^2^ were used to identify and convert metabolites.

### Metabolite Prediction Using Mangosteen

A relationship table linking KEGG orthologs (KO) and BioCyc reactions (RXNs) to the compounds housed in KEGG (release date 2019 May) and BioCyc (version 23.0) databases was compiled. Specifically, if KOs have corresponding KEGG reactions which produce or consume KEGG compounds, we directly linked those KOs to KEGG compounds. An example is that K24443 corresponds to two reactions R02428, R02526, which produce or consume compounds C02753, C00001, C00502, C01114, and C00545. Thus, K24443 is linked to those compounds. There are cases when KOs do not have corresponding reactions but KEGG Enzyme numbers, and we would link those enzymes to reactions and further to compounds. An example is that K00046 has no corresponding reactions but the enzyme number EC:1.1.1.69, through which we linked to reactions R01738, R01740 and the reactions produce or consume compounds C00257, C00003, C01062, C00004, C00080, C00006, C00005, thus those compounds are linked to K00046. Manual effort was also made to link transporter KOs (BRITE class: ko02000) to their associated compounds. For the BioCyc database, RXNs and their associated compounds were inferred from “core_description_of_RXNs” directly, which included reactions from both MetaCyc and all PGDBs in BioCyc. This collection of reactions is referred to as “BioCyc reactions” in the remainder of the text. Reaction directionality was not taken into consideration when constructing the relationship table because many biochemical reactions are reversible depending on the reactant and product concentrations.

We inferred all metabolites that linked to KOs or RXNs based on the relationship table for coverage evaluation. If a KO or RXN links to multiple metabolites, all were reported. For differential metabolite prediction, we first identified differential microbial functions (KOs/RXNs) between case and control samples *via* DESeq2 analysis (adjusted *p*-value threshold = 0.2; Love et al., 2014) following prevalence filtering at 5%. The list of differential microbial functions was used as the input for Mangosteen and all compounds linked to them were reported to be differential compounds.

### Metabolite Prediction Using MIMOSA

Using the source code from MIMOSA^3^, we updated the community metabolic network template to the May 2019 version of the KEGG database. Microbiome functional abundance data were normalized by means of MUSiCC using default settings (Manor and Borenstein, 2015), as is recommended prior to applying MIMOSA for prediction. We then calculated the community-metabolic-potential (CMP) of each sample, which served as the predicted metabolome.

### Metabolite Prediction Using MelonnPan

In lieu of applying the default model included in the MelonnPan package (Mallick et al., 2019), we constructed a model based on metabolome and microbial function abundance data from six previous investigations (Table 1). Because multiple microbiome sequencing datatypes exist for two studies (shotgun metagenomic and metatranscriptomic data in Lloyd et al. study; Lloyd-Price et al., 2019; 16S rRNA gene amplicon and metatranscriptomic data in SG_IBD study), we chose to leave-one-study-out cross-validation to minimize potential overfitting for MelonnPan. We retained all metabolites and microbial KOs or RXNs with over 10% prevalence and mean relative abundance over 0.01% in accordance with MelonnPan operating procedures. From the model that resulted, we mitigated the potential for spurious results by omitting metabolites predicted by only one KO or RXN from consideration (model size = 1).

### Evaluation Metric

Predicted community metabolite profiles were compared to empirically measured metabolite profiles, and both occurrence and abundance were appraised. We then calculated the precision, recall, and F1 score for each dataset per pipeline. Occurrence evaluation only considers the presence or absence of a metabolite in a given microbial community. Abundance evaluation considers both the results of Procrustes analyses conducted across the predicted and measured abundance matrices (Peres-Neto and Jackson, 2001) and the identification of differential metabolite described below.

Prevalence filtering was applied to measured metabolome data, as well as MIMOSA and MelonnPan predicted metabolome data, and metabolites detected or predicted in fewer than 5% of the samples were omitted from consideration. Data resulting from empirically measured- and MelonnPan predicted-profiles were log transformed after imputing zeros with the minimum non-zero value per metabolite. We then conducted Student’s *t*-tests to identify differential metabolites between case and control samples, followed by Benjamini-Hochberg FDR correction (adjusted *p*-value threshold = 0.2). As the abundance matrix generated with MIMOSA includes both positive and negative values, we elected to apply Wilcoxon rank sum tests with Benjamini-Hochberg FDR correction (adjusted *p*-value threshold = 0.2). Wilcoxon signed rank tests were used to comparatively evaluate precision, recall, and F1 scores across pipelines and databases between paired datasets.

Random sampling analysis was also performed and compared to the pipeline prediction. For coverage evaluation, the same number as the predicted metabolites were randomly selected from the in-house chemical dictionary and compared with the pipeline predicted results. For differential metabolite identification, gene labels were randomly shuffled prior to DESeq2 and used for prediction for Mangosteen. For MIMOSA and MelonnPan, predicted metabolite labels were shuffled in the predicted matrices, followed by the same procedure for differential metabolite identification. The results were further evaluated against the measured metabolites and performance was represented in precision, recall and F1 score. The procedure was repeated for 99 times.

## Results

### Selected Studies Include Diverse Metabolome and Microbiome Sequencing Technologies

Among the 6 studies that fit our selection criteria as described in Supplementary Figure 1, we considered the datasets from three IBD studies (Franzosa et al., 2019; Lloyd-Price et al., 2019), one colorectal cancer (CRC) investigation (Hale et al., 2018), one autism spectrum disorder study (Kang et al., 2018), and one dietary intervention (Maier et al., 2017; Table 1). Many of the samples originated from stool (*n* = 648 from five studies), while a smaller subset (*n* = 83) arose from intestinal tissue samples of the CRC study. All samples were subjected to metabolomic analysis *via* either mass spectrometry (MS; five studies) or NMR spectroscopy (NMR; one study). For microbiome profiling, half of the datasets originate from 16S rRNA gene amplicon sequencing (four datasets), followed by shotgun metagenomic analysis (two datasets), and metatranscriptomic sequencing (two datasets).

### Pipeline Paradigm Determines Metabolite Prediction

Empirically measured metabolome profiles from each of the aforementioned datasets were compared directly with pipeline predictions. Only peaks corresponding to known compounds were retained, which resulted in a total of 1,998 metabolites spanning six studies. We observed significant differences in metabolite numbers across different metabolomics technologies. Fourier transform ion cyclotron resonance mass spectrometer (FT-ICR-MS) identified the greatest number of metabolites (*n* = 1,273) and NMR the least (*n* = 62; Supplementary Figure 2A). Of all metabolites detected, 1,887 corresponded to KEGG-specific compound identifiers and 987 to BioCyc-specific compound identifiers. Quality-controlled metagenomic and metatranscriptomic data were mapped against KEGG and BioCyc databases and Piphillin (Iwai et al., 2016; Narayan et al., 2020) was applied to 16S rRNA sequencing data to infer communities’ functional profiles. Ultimately, we identified 11,527 KOs and 8,427 RXNs across eight datasets spanning six studies (Supplementary Figure 2B), which collectively served as the input for each prediction pipeline.

**FIGURE 2:**
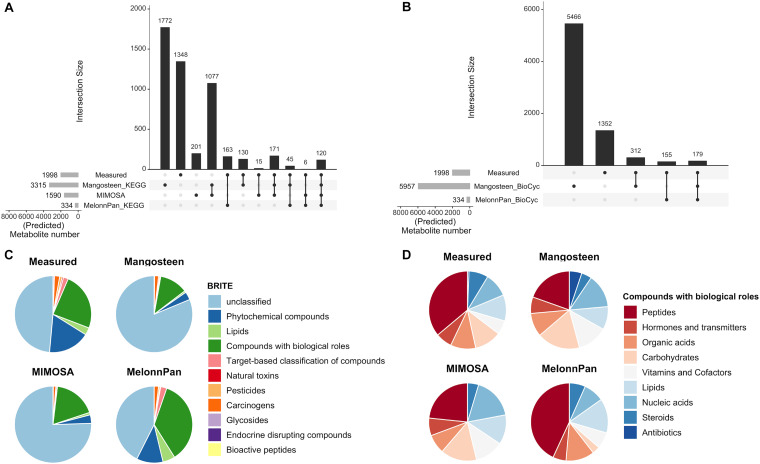
Results of metabolite prediction as performed by different pipelines. Upset plots (Lex and Gehlenborg, 2014) depict the measured and predicted metabolite numbers resulting from each pipeline and their intersections based on **(A)** KEGG and **(B)** BioCyc databases. Pie charts display predicted metabolite classification according to **(C)** KEGG BRITE classes, and specifically **(D)** metabolites belonging to the “Compounds with biological roles” BRITE class.

The Mangosteen pipeline identified 3,315 KEGG- and 5,957 BioCyc-associated metabolites across all studies (Magosteen-K and Mangosteen-B, respectively). While Mangosteen-B predicted significantly fewer unique microbial functions than Mangosteen-K (Supplementary Figure 3A), it linked to significantly more metabolites (Supplementary Figure 3B). MIMOSA predicted a total of 1,590 metabolites using the KEGG database, 1,077 of which were shared with Mangosteen-K (Figure 2A). In comparison, the ML-based MelonnPan pipeline predicted only 334 metabolites upon interrogating models built on microbial functions mapped to KEGG (MelonnPan-K) or BioCyc (MelonnPan-B; Figures 2A,B and Supplementary Figure 4).

Metabolites bearing KEGG identifications were then assigned to their corresponding BRITE classifications (Kanehisa et al., 2011). Melonnpan-K predicted significantly greater fractions of metabolites deemed “Compounds with biological roles” (BRITE ID: 08001), “Phytochemical compounds” (08003), “Lipids” (08002), and “Target-based classification of compounds” (08010) than the reference-based approaches (Figure 2C). Considering only the “Compounds with biological roles” category, MelonnPan-K predicted the greatest fractions of metabolites resembling “Peptides,” “Organic acids,” “Lipids,” and “Steroids” and smallest fractions of those resembling “Nucleic acids,” “Vitamins and Cofactors,” “Hormones and transmitters,” and “Carbohydrates” (Figure 2D). Mangosteen-K was the only pipeline to predict metabolites belonging to the “Antibiotics” category of classification.

### ML-Based MelonnPan Yielded the Highest F1 Score in Predicting Metabolite Occurrence

**FIGURE 3:**
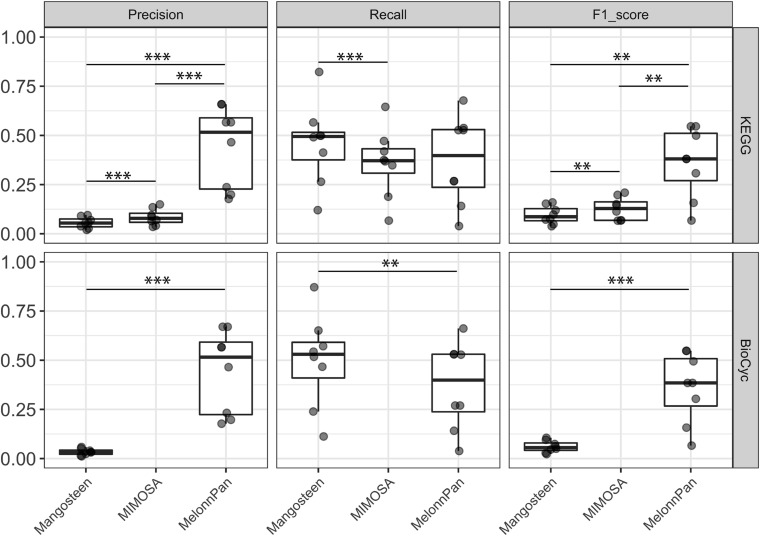
Evaluation of predicted occurrence (presence/absence) as appraised by precision, recall, and F1 score. Each point indicates a dataset used for evaluation. A pairwise Wilcoxon signed-rank test was applied at ^∗∗∗^*p* < 0.01, ^∗∗^*p* < 0.05.

Predicted metabolite profiles were compared to empirically measured metabolite profiles. MelonnPan exhibited the greatest precision (MelonnPan-K: mean 0.44, MelonnPan-B: mean 0.44) in predicting the presence/absence of metabolites in the human gut, followed by MIMOSA (0.08) and Mangosteen (Mangosteen-K: 0.06, Mangosteen-B: 0.03; Figure 3). Recall was significantly greater with Mangosteen-K, as opposed to MIMOSA, and with Mangosteen-B as opposed to MelonnPan-B. Taking into consideration both precision and recall, F1 scores demonstrated the superiority of ML-based MelonnPan predictions (MelonnPan-K: 0.36, MelonnPan-B: 0.36). KEGG-referenced Mangosteen exhibited a small but significant increase in both precision (Mangosteen-K vs. Mangosteen-B: mean 0.06 vs. 0.03, *p* = 0.01) and overall F1 scoring (0.1 vs. 0.06, *p* = 0.01) compared to its BioCyc-referenced counterpart. With respect to ML-based MelonnPan, these two reference databases used for model building performed equally well and yielded no significant differences in precision, recall, or F1 score. We also compared the prediction results to random sampling, which randomly predicted the same number of metabolites, and all pipelines showed significantly better performance regarding precision, recall and F1 score (*p* < 0.01).

### None of the Predicted Metabolite Profiles Retain High Levels of Similarity to the Empirically Measured Metabolome

As both MIMOSA and MelonnPan output metabolite abundance predictions, we examined the extent of similarity between the predicted and empirically measured metabolite abundance profiles. Euclidean distance from the predicted and measured metabolomes were calculated to facilitate Procrustes analyses (Peres-Neto and Jackson, 2001). Very little similarity was observed between the metabolomes (correlation coefficient range: 0.04–0.26, *p* > 0.05), with squared m12 values (a measure of fit between two datasets; low value indicates high similarity; Peres-Neto and Jackson, 2001) ranging from 0.93 to 1 (Supplementary Figure 5). With respect to Procrustes analysis, MIMOSA- and MelonnPan-generated prediction profiles did not significantly differ when compared to measured metabolome, and neither did KEGG-referenced and BioCyc-referenced MelonnPan predictions.

### All Pipelines Performed Poorly in Identifying Differential Metabolites

**FIGURE 4:**
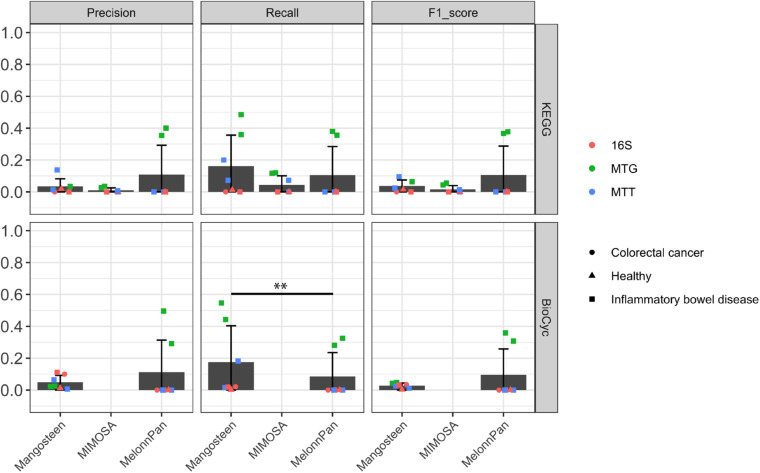
Evaluation of predicted differential metabolite identification as appraised by precision, recall, and F1 score. Each point indicates a dataset used for evaluation. A pairwise Wilcoxon signed-rank test was applied at ^∗∗^*p* < 0.05.

We evaluated these pipelines on their ability to identify differentially abundant metabolites between case and control groups as shown in Table 1. The predicted differential metabolites identified in each pipeline were compared to differential metabolites identified in experimental data. ML-based MelonnPan predictions yielded the highest precision and F1 scores (but still low and not significant) compared to the two reference-based prediction methods (MelonnPan-K Precision mean: 0.11, F1 score: 0.11; MelonnPan-B: Precision: 0.11, F1 score: 0.10; Figure 4). We also compared the results to random sampling, where differential metabolites were identified from either shuffled microbial abundance table (for Mangosteen) or predicted metabolite abundance tables (for MIMOSA and MelonnPan). Despite of the overall low F1 score, Mangosteen showed significantly higher F1 scores (*p* < 0.05) compared to random prediction while MIMOSA and MelonnPan did not (MIMOSA: *p* = 0.2, MelonnPan-K: *p* = 0.37, and MelonnPan-B: *p* = 0.42).

## Discussion

Gaining insight into the metabolite pool of a microbial community is of paramount importance to understand its ecological role(s), not to mention its potential as a source of therapeutics and other invaluable molecules (Jia et al., 2008; Patel and Ahmed, 2015; Wishart, 2016). As an alternative to cost- and resource-prohibitive full-scale metabolome profiling, metabolome prediction from *a priori* microbiome sequencing data affords researchers the ability to generate hypotheses in a rapid, cost-effective, and relatively reliable fashion. We systematically compared the ability of metabolite predictions by two published tools alongside a newly developed reference-based pipeline using more than 900 paired microbiome-metabolome stool/intestinal tissue samples from six different studies on various human diseases. Resulting metabolite profile predictions were compared to one another and contrasted alongside an empirically measured metabolome dataset, focusing primarily on occurrence (i.e., presence vs. absence) and identification of differentially abundant metabolites.

When used to predict the presence or absence of given metabolites within a community, the ML-based MelonnPan approach significantly outperformed its reference-based competitors, with respect to overall precision and F1 score. However, the reference-based Mangosteen and MIMOSA pipelines did exhibit high recall despite their lower precision and F1 scores. As recall is calculated based on true-positive and false-negative tallies, the high number of metabolites predicted *via* these reference-based pipelines likely contributed to the elevated recall values. In addition, any given KO may participate in any number of distinct reactions, bearing the potential to dramatically increase the number of associated metabolites. With regard to reference-based methods, MIMOSA surpassed Mangosteen-K in both precision and F1 score. This speaks to the benefit of considering the directionality as well as the network of connections between reactions within a community. Although Mangosteen showed worse performance in metabolite prediction compared to MIMOSA, it was able to link more metabolites and could be useful for mining metabolites outside of known metabolic network. While the type of microbiome data examined also affects prediction performance, we lacked an appropriate number of independent datasets to evaluate this aspect in our study (one study for metagenomic to metatranscriptomic and another for 16S rRNA amplicon to metatranscriptomic sequencing comparisons). A pipeline’s ability to accurately distinguish differential metabolites between case and control groups is typically a sound foundation from which hypotheses are structured and biomarker and therapeutics discovery initiatives expound. Such evaluations are predicated upon accurate predictions of metabolite abundance, and as such, are much more stringent than occurrence prediction. While we did not observe promising results in any of the pipelines, MelonnPan predictions yielded the highest mean F1 scores (around 0.1 in both MelonnPan-K and MelonnPan-B).

Machine-learning-based MelonnPan outperformed the reference-based methods in predicting both occurrence and differential metabolites. In contrast to the reference-based pipelines, this powerful technique considers the impact of host metabolism and hitherto undiscovered interactions that do not exist in reference databases. Like all ML strategies, MelonnPan relies heavily on the accuracy of training data. As such, while untargeted metabolome profiling strategies generate the most comprehensive overview of the metabolites present within a sample, coverage is unavoidably limited due to the inherent technical biases associated with either MS or NMR (Christians et al., 2011; Vuckovic, 2012). Hence, we were mindful to include metabolome data generated by multiple platforms to expand and diversify the training set and thus improve coverage of human gut-associated metabolites. In the current evaluation, although we tried our best to include as many paired samples as possible (over 900 pairs) to train the ML model, variance in prediction performance was observed across studies, which suggests more training sets are still needed to obtain a general and robust prediction model.

While conducting the differential metabolite evaluation, we observed the highest F1 score in the MelonnPan-predicted metagenomic dataset of Franzosa et al. (2019) as well as the metagenomic dataset of Lloyd-Price et al. (2019). Similar results were observed while evaluating MelonnPan with datasets generated in three independent IBD studies (for a disease-specific model; Supplementary Figure 6). Of all the datasets evaluated, these two were generated in the most similar manner, i.e., metabolome data were obtained from 4 LC-MS methods (targeting polar metabolites, metabolites of intermediate polarity, and lipids) while microbiome metagenomic sequencing data were generated using an Illumina HiSeq platform. Numerous factors and processes contribute significantly to variances observed between studies (e.g., sample preparation, instrument settings, and user variation) in both metabolome and microbiome sequencing-based investigations (Gika et al., 2010; Tulipani et al., 2013; Clooney et al., 2016). Thus, the high F1 scores observed in Franzosa et al. and Lloyd-Price et al’s metagenomic datasets are likely due to similar data generation techniques. Furthermore, it stands to reason that while one dataset is used in a training model, prediction for a new study of similar datatypes is more accurate. This could also explain the superior performance reported in Mallick et al. (2019) MelonnPan paper, wherein training and testing datasets were generated using the exact same microbiome sequencing and metabolomic technologies. We also noticed metatranscriptomic-based prediction performed worse than metagenomic-based prediction for differential metabolite identification. This again could be due to the dataset type in the training set because we observed higher RTSI scores (a measurement to quantify the representativeness of new samples with respect to training datasets from MelonnPan; the higher the value, the more accurate prediction is; Mallick et al., 2019) when using metagenomic data for prediction compared to metatranscriptomics in the Lloyd-Price et al’s study. The choice of training datasets will also impact the computing demand for ML-based methods and should be taken into consideration. From our experiences, MelonnPan took average 24 CPU hours to train with the current datasets (∼900 paired samples) while the prediction step finished in less than 10 min, similar to the reference-based MIMOSA and Mangosteen prediction. Ultimately, the training time could be eliminated if one general model is built and used repeatedly for prediction. This information is extremely useful for future metabolite prediction applications using ML-based approaches like MelonnPan and reminds the user of the importance of choosing appropriate training and prediction datasets with the goal of optimizing downstream prediction indices. With more and more metabolomic data being deposited into public domains, the ability to construct either datatype depend or independent ML models will expand rapidly and researchers will be able to choose appropriate datasets for training and prediction. Moreover, advances in untargeted metabolomic technologies with broader coverage, superior resolution, and greater accuracy (Ghaste et al., 2016; Ortmayr et al., 2016; Týčová et al., 2017; Bingol, 2018) will collectively lead to significantly improved training data, and in turn, more accurate ML-based predictions.

As opposed to ML-based and data-driven approaches, reference-based methods can potentially identify/predict metabolites whose concentration or specific chemical properties precluded them from detection *via* conventional untargeted metabolome profiling as well as ML-based prediction (Karu et al., 2018). Reference-based prediction pipelines make use of well-curated information in massively large repositories to infer possible metabolites within a sample. As such, the integrity and comprehensiveness of the database to be queried factors significantly into the process. KEGG (Kanehisa et al., 2011) and BioCyc (Caspi et al., 2011) are two widely used databases that house curated metabolic pathway information from the annotated genomes. With regard to Mangosteen predictions, we linked significantly more metabolites from RXNs collected from BioCyc than the KEGG database (Supplementary Figure 3), but this did not help BioCyc-based prediction to achieve greater F1 scores. The greater number of observed compound connections with BioCyc is consistent with previous reports showing BioCyc houses a greater number of compounds associated with reactions (“all reaction substrates”) than KEGG (Altman et al., 2013). There are also many assumptions germane to reference-based prediction that are not accurate when describing the human gut microbiome (e.g., gene and/or transcript abundance is reflective of enzymatic activity, microbial communities are well-mixed, steady-state systems *sans* compartmental effects; Kumar et al., 2019). Nevertheless, *de novo* metabolite prediction is only one possible use of these reference-based tools, as the full functionality of MIMOSA is to identify metabolites which show strong evidence of their microbial producers/consumers and Mangosteen is better at identifying all linked metabolites when given a microbial feature (KO or RXN), either differential ones between case and control or any features the researchers are interested in. Ultimately, caution and common sense go a long way in interpreting the predicted metabolite profiles resulting from reference-based pipelines.

There are several limitations in the current evaluation that need to be taken into consideration when researchers try to choose the optimal prediction tool for their research. While we treated the empirically measured, untargeted metabolome as a “gold standard” and directly compared all pipeline prediction results to this profile, biases in technology render even this portrayal imperfect in its attempt to unveil the true and complete metabolite pool (Christians et al., 2011; Vuckovic, 2012; Karu et al., 2018). In reality, predicted metabolites that were treated as false positives might not actually be false, and in turn, the actual performance of these prediction pipelines might supersede what we report here. Additional profiling of these “false positive” metabolites or technological advances in untargeted metabolomics will help to mitigate the issue on “false positive” predictions. In addition, although we tried to select the metabolome data in a uniform way, there are variations in extraction methods, chromatography conditions, model of the machines as well as software and databases between studies (Supplementary Table 1), which are currently not considered during evaluation. Secondly, the current evaluation only focuses on the human microbiome from stool and intestinal tissues, the performance of these software on other types of microbiome data, such as environmental samples, could vary and warrants further evaluation efforts. Moreover, emerging tools that integrate microbiome and metabolome data could add more interpretability beyond metabolites prediction and help researchers to understand the origin of metabolites and its association with microbiome, such as Annotation of Metabolite Origins via Networks (AMON; Shaffer et al., 2019), neural network based mmvec (Morton et al., 2019), MIMOSA2 (MIMOSA2, 2020), Generalized correlation analysis for Metabolome and Microbiome (GRaMM; Liang et al., 2019), etc.

Predicting metabolic capacity and metabolite diversity is of paramount consequence to better understanding and manipulating the function(s) of microbial communities, all of which bears immense significance to improving human health and empowering environmental microbiome research (Turnbaugh and Gordon, 2008; van Dam and Bouwmeester, 2016; Van Treuren and Dodd, 2020). Ultimately, this evaluation serves as a framework and launching point for future initiatives interested in exploiting metabolite prediction as a cost-effective means of generating cogent hypotheses.

## Data Availability Statement

Metadata, 16S rRNA gene amplicon and metatranscriptomic sequencing data generated in this study (SG_IBD) have been deposited in the NCBI database under BioProject ID PRJNA668188.

## Ethics Statement

The studies involving human participants were reviewed and approved by University of Calgary, Conjoint Health Research Ethics Board (ID:18142 and 14-2429). The patients/participants provided their written informed consent to participate in this study.

## Author Contributions

SI, TD, and KD conceived and supervised the project. XY, TA, TD, and SI designed the study. XY, ER, KW, YW, JC, PB, and GK performed data collection and processing. XY conducted the analysis and wrote the first draft of the manuscript. All authors edited and approved the manuscript.

## Conflict of Interest

XY, ER, KW, YW, JC, KD, TD, and SI are employees of Second Genome Inc., which provides salaries and stock options. Second Genome Inc. is an independent therapeutics company with products in development to treat intestinal disorders and other human diseases. TA is an employee of Altman Analytics LLC. The remaining authors declare that the research was conducted in the absence of any commercial or financial relationships that could be construed as a potential conflict of interest.

## Footnotes

1 https://pubchem.ncbi.nlm.nih.gov/idexchange/idexchange.cgi

2 https://cts.fiehnlab.ucdavis.edu/batch

3 https://github.com/borenstein-lab/MIMOSA
