# Supplementary material for: A Comparative Evaluation of Tools to Predict Metabolite Profiles From Microbiome Sequencing Data
Source: Front Microbiol. 2020 Dec 4;11:595910. doi: 10.3389/fmicb.2020.595910 (PMC7746778; doi:10.3389/fmicb.2020.595910)
Supplement: Supplementary file 1 [file Data_Sheet_1.docx]

Supplementary Material

A comparative evaluation of tools to predict metabolite profiles from microbiome sequencing data

Xiaochen Yin, Tomer Altman, Erica Rutherford, Kiana A. West, Yonggan Wu, Jinlyung Choi, Paul L. Beck, Gilaad G Kaplan, Karim Dabbagh, Todd Z. DeSantis & Shoko Iwai^*^

*** Correspondence:**Shoko Iwai
shoko@secondgenome.com

# Supplementary Figures

**Supplementary Figure S1.**  Search and filtering criteria for microbiome-metabolome paired datasets.

**
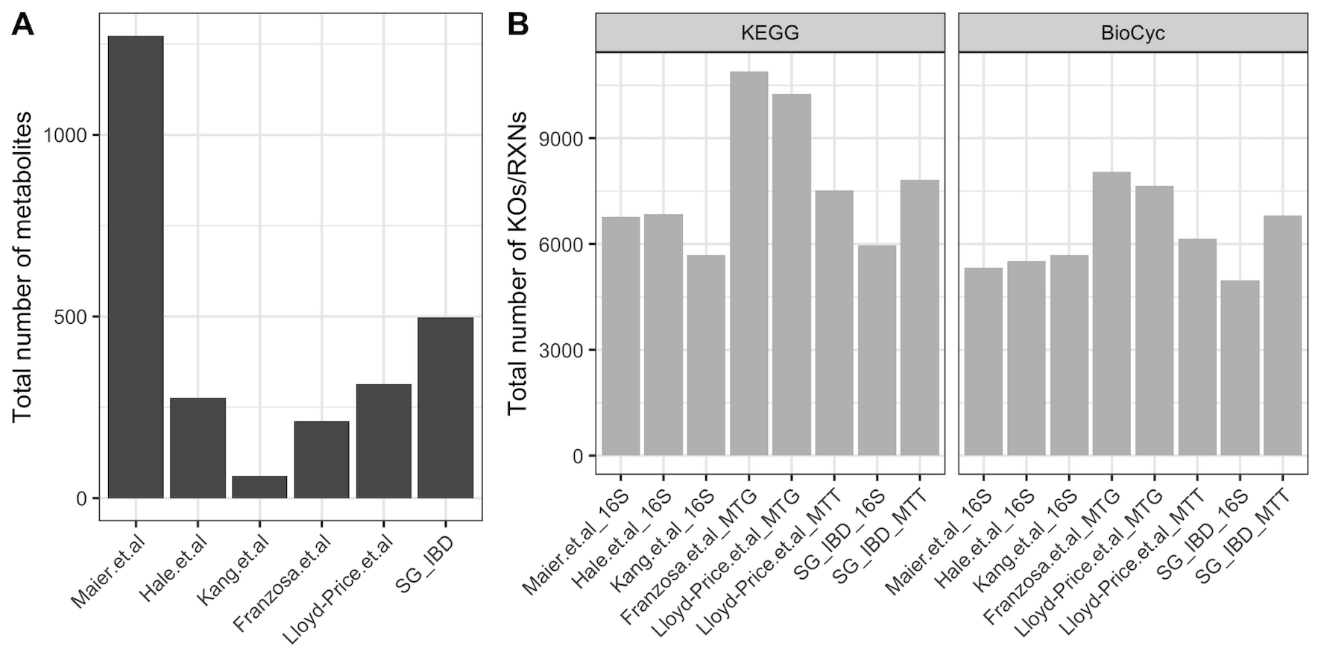
Supplementary Figure S2.** The number of (**A**) metabolites assigned with unique in-house identifiers and (**B**) microbial functions (KOs/ RXNs) housed in each dataset used for metabolite prediction and evaluation. 16S, 16S rRNA gene amplicon sequencing; MTG, shotgun metagenomic sequencing; MTT, metatranscriptomic sequencing.


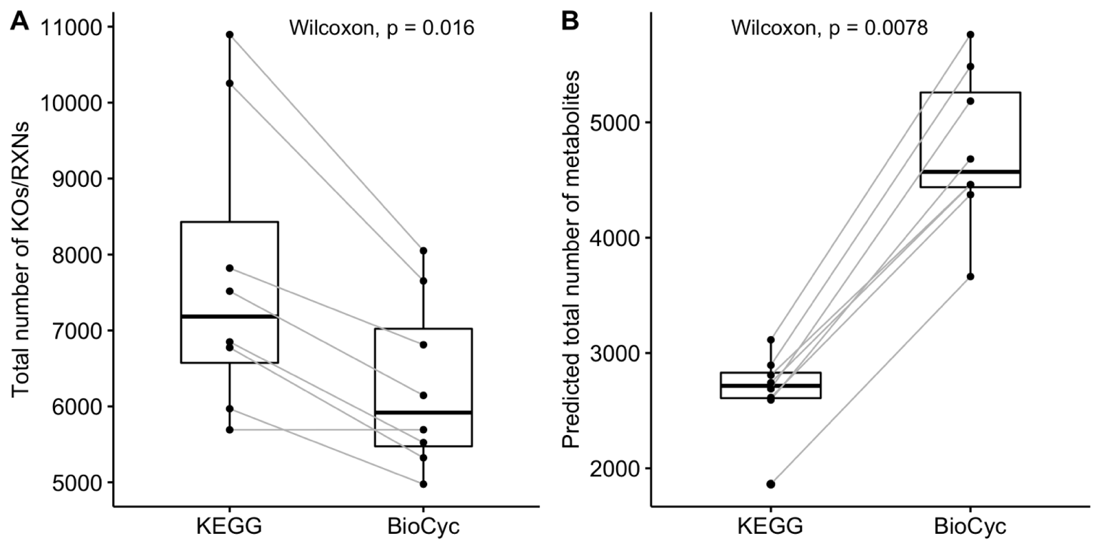


**Supplementary Figure S3**. The number of (**A**) mapped or inferred microbial functions (KOs/ RXNs) and (**B**) predicted metabolites using the Mangosteen pipeline with the KEGG and BioCyc databases. Wilcoxon signed-rank test was applied.


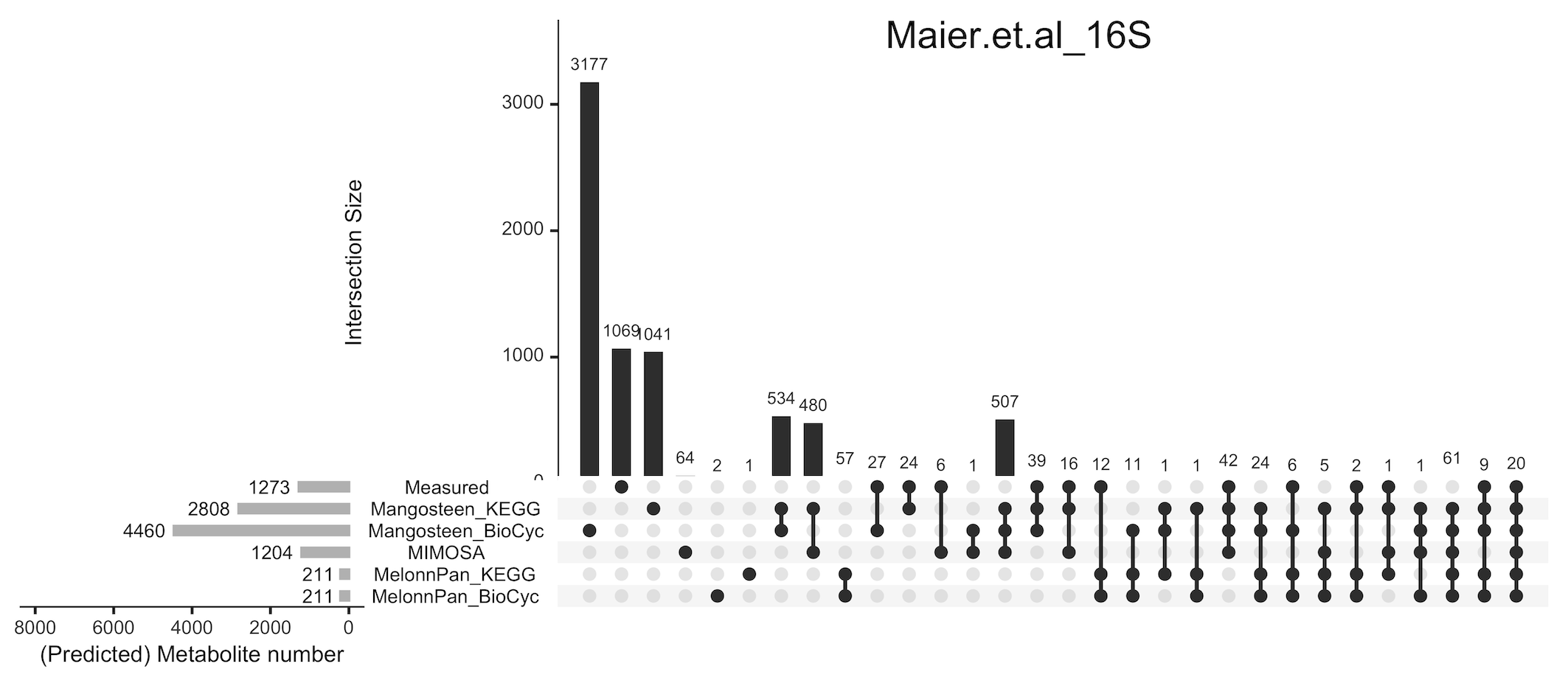

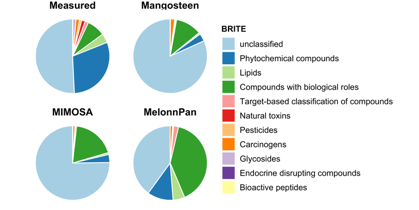

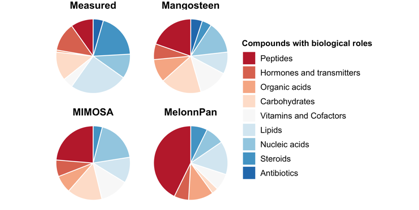

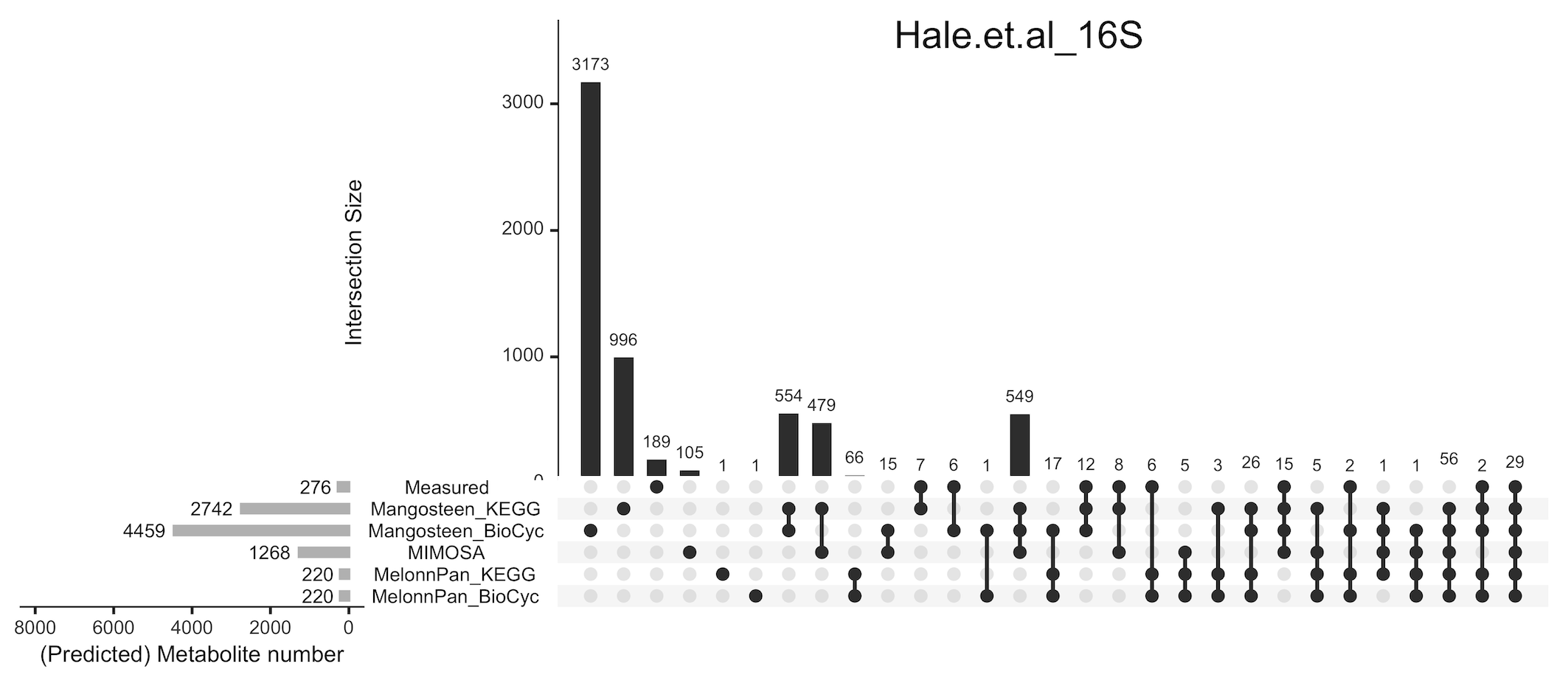

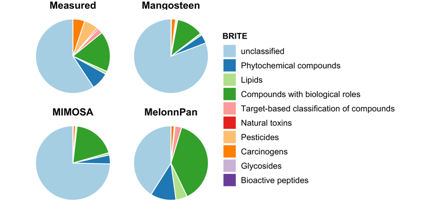

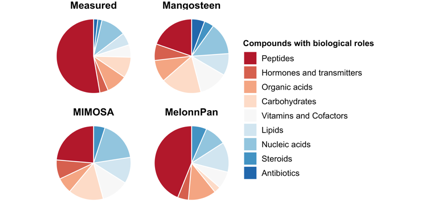

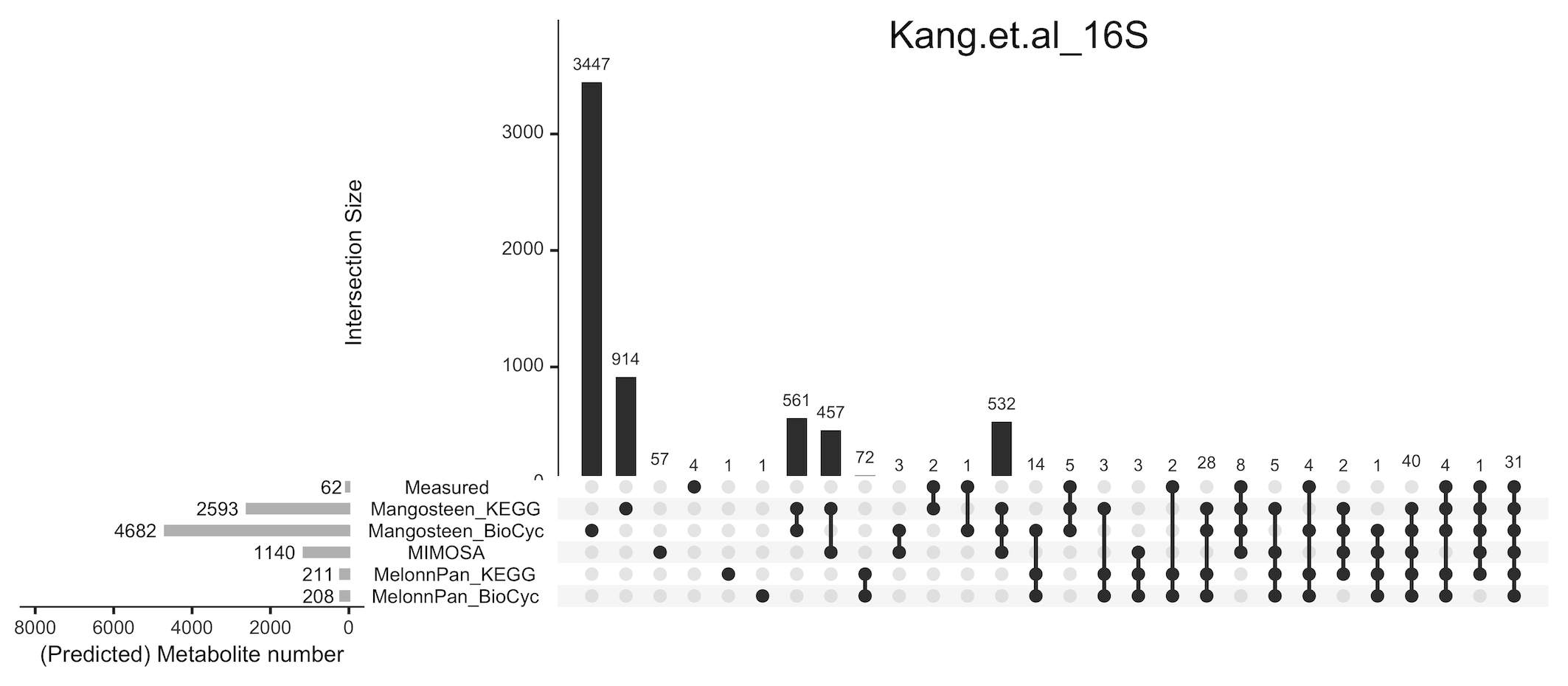

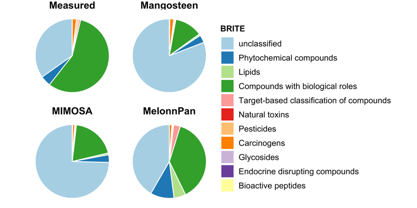

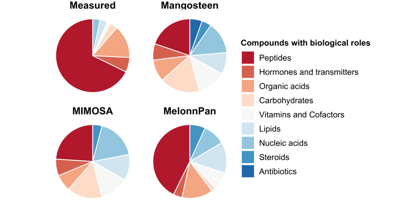

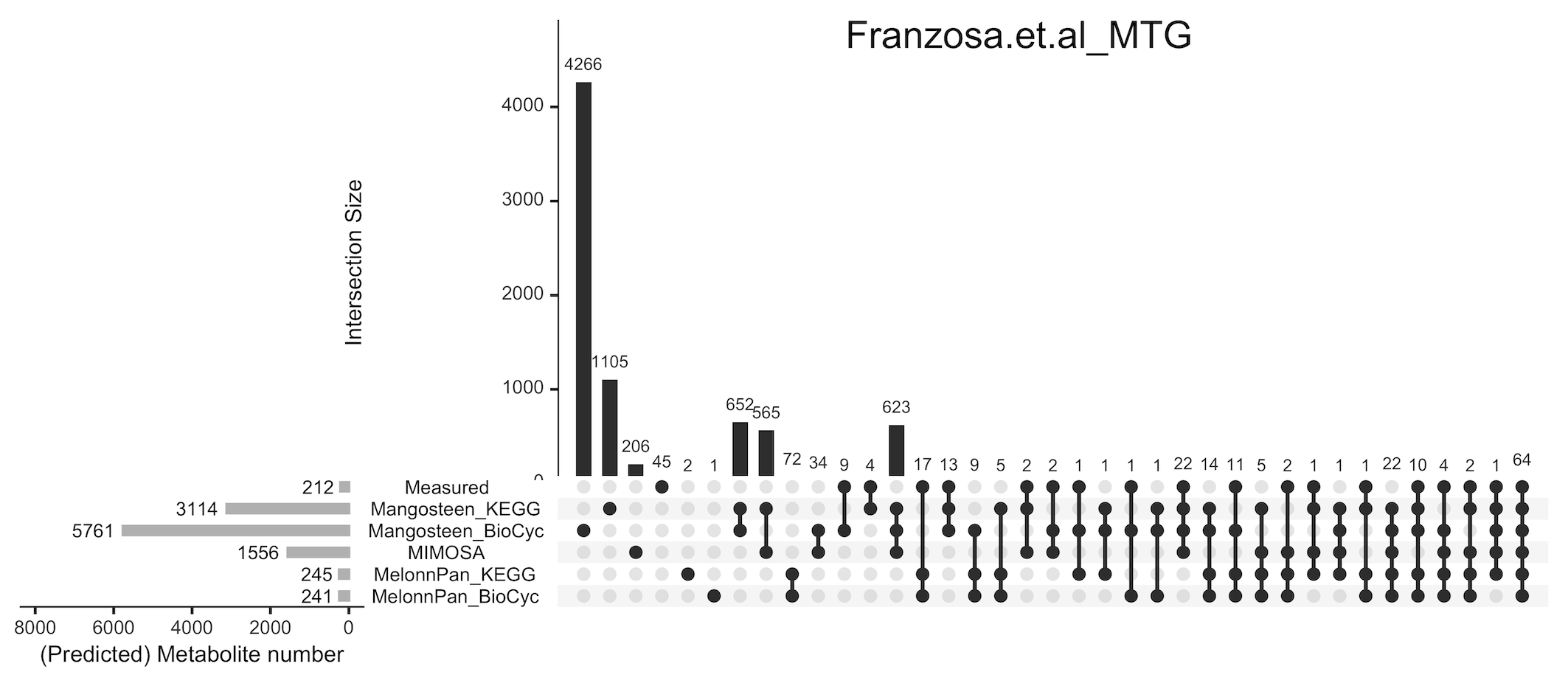

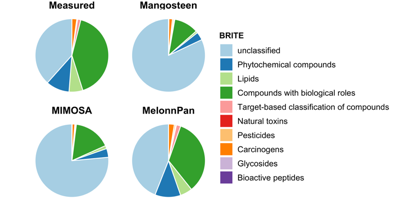

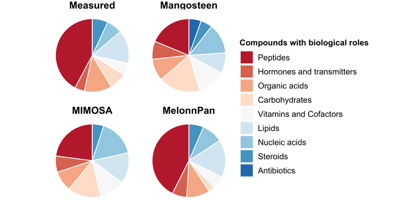

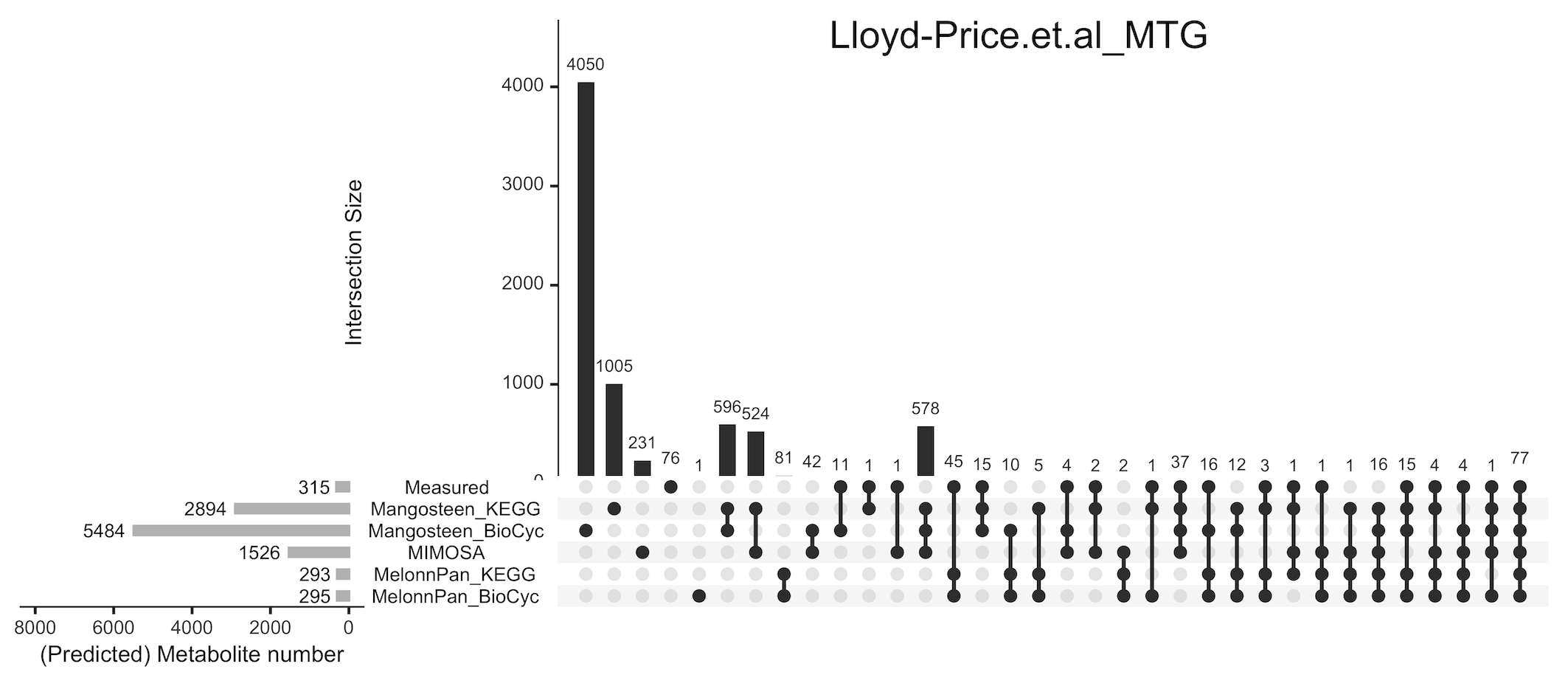

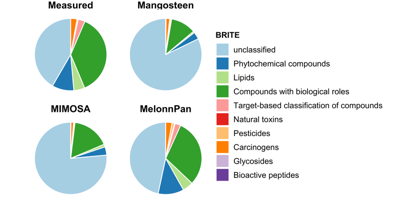

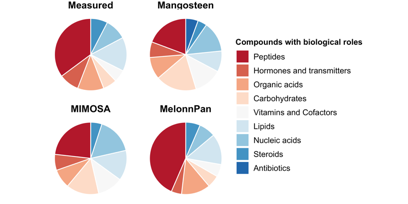

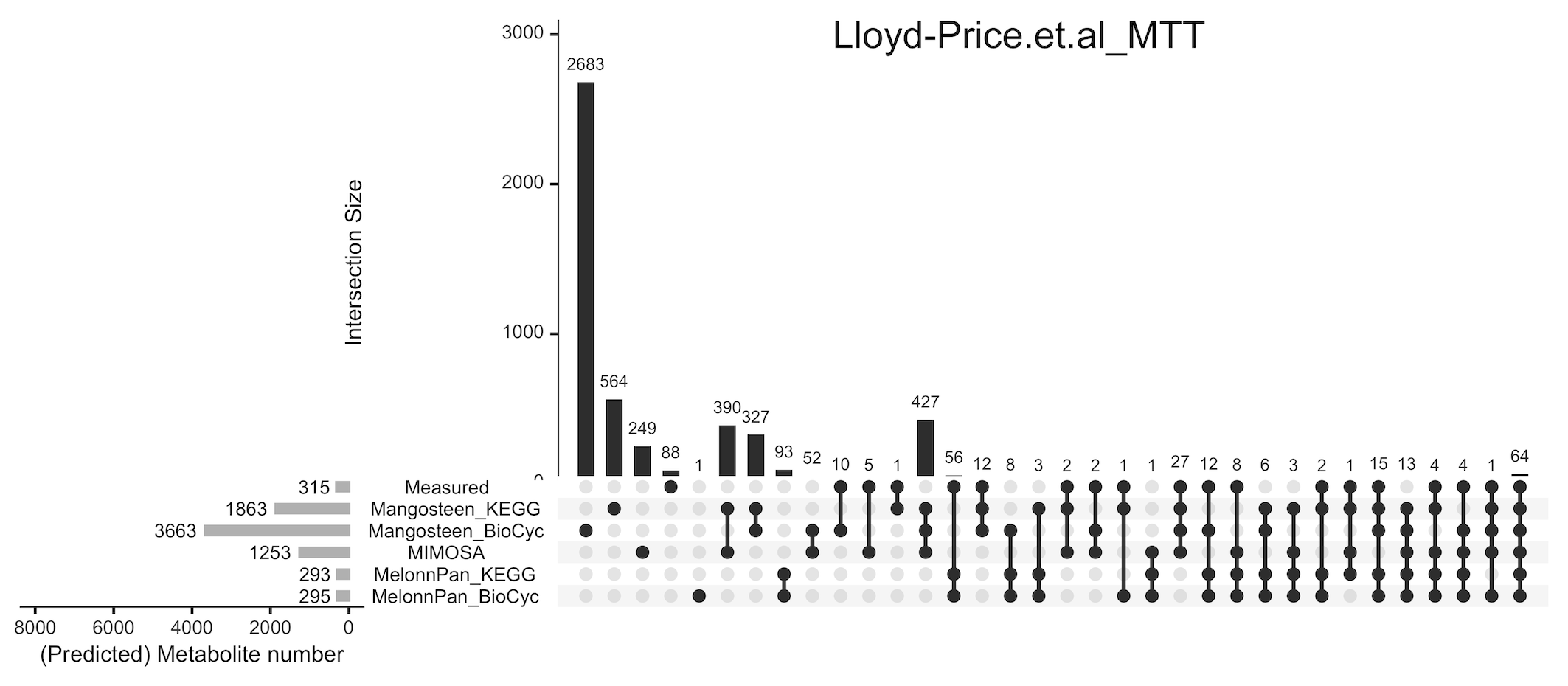

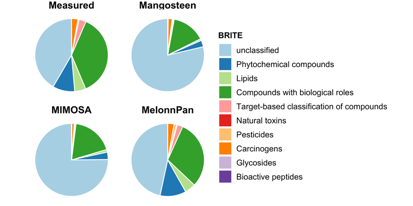

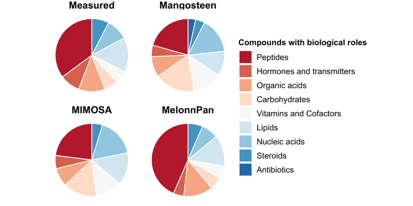

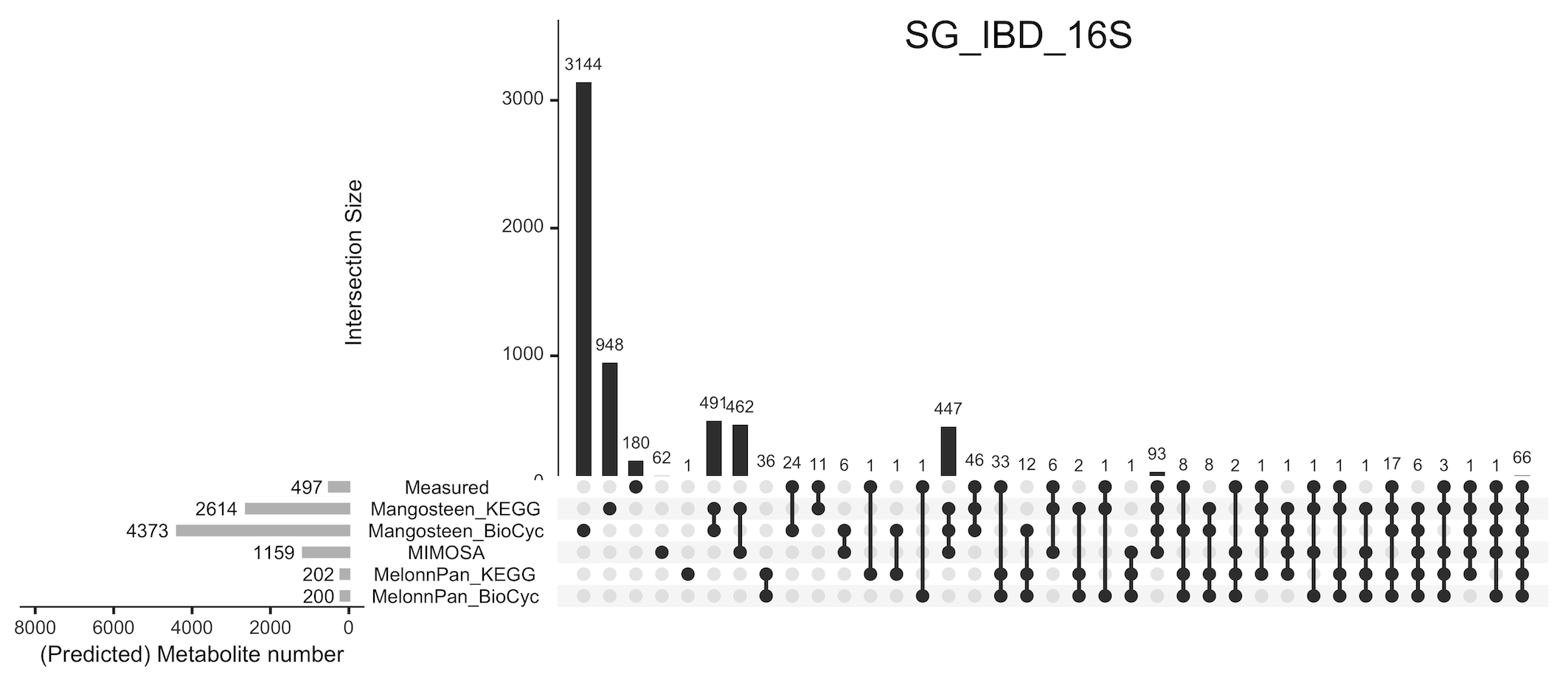

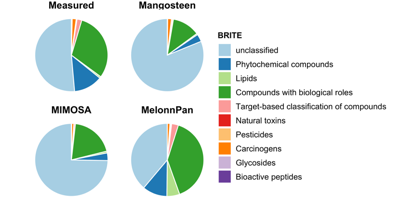

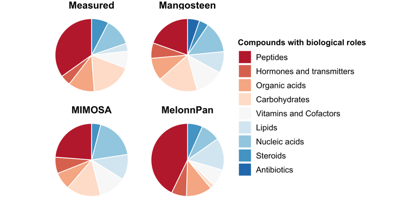

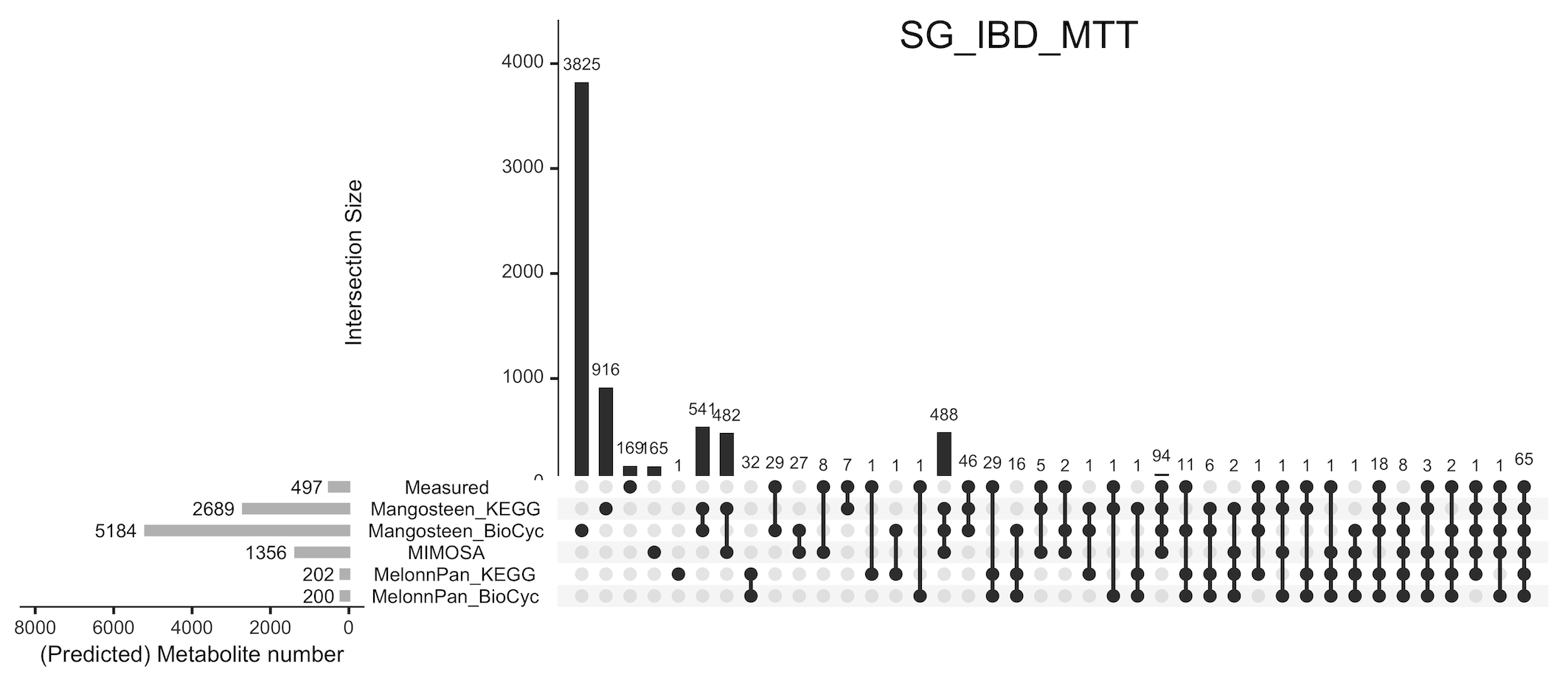

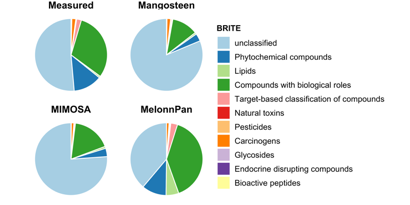

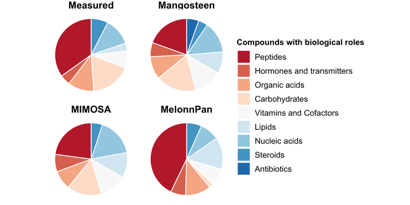


**Supplementary Figure S4**. Results of metabolite prediction for each dataset from different pipelines. Upset plots depict the measured and predicted metabolite numbers resulting from each pipeline and their intersections based on KEGG and BioCyc databases. Pie charts display predicted metabolite classification according to KEGG BRITE classes (left), and specifically metabolites belonging to the “Compounds with biological roles” BRITE class (right). The title of each plot is the combination of study name and microbiome sequencing type (**Table 1**). 16S, 16S rRNA gene amplicon sequencing; MTG, shotgun metagenomic sequencing; MTT, metatranscriptomic sequencing.


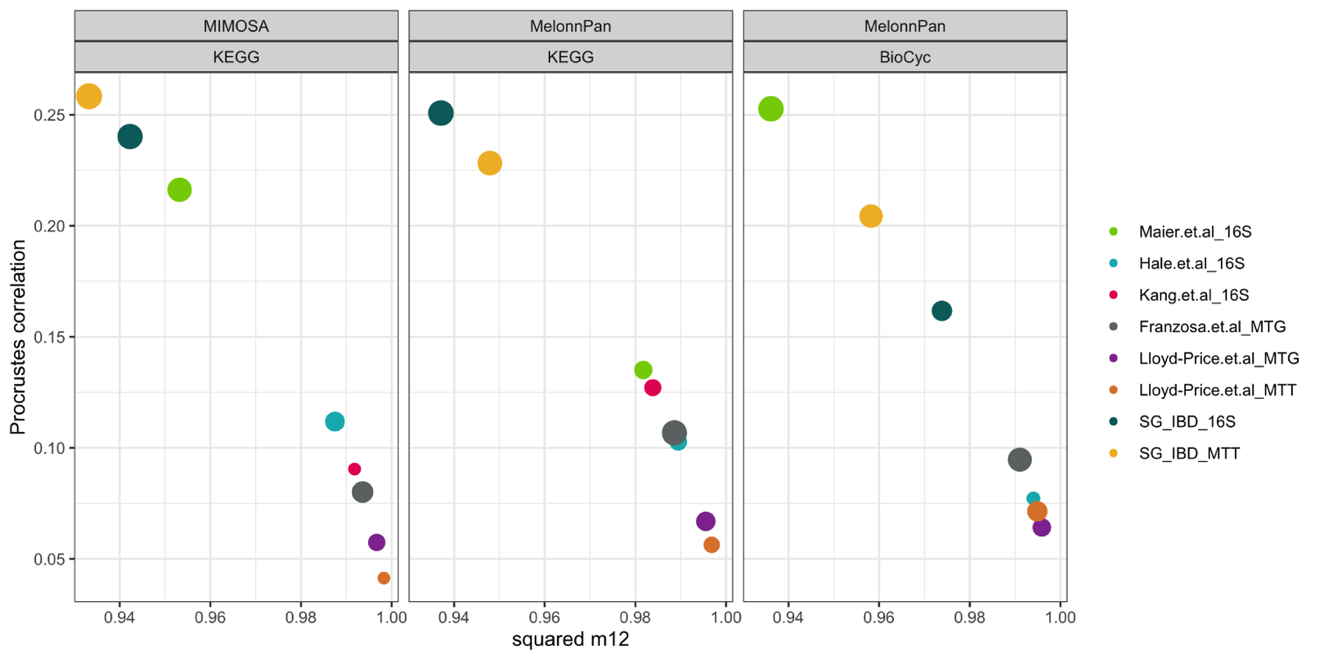


**Supplementary Figure S5**. Procrustes analysis between measured and predicted metabolome abundance data using MIMOSA and MelonnPan with 999 permutations. The relative size of the points correlates inversely with the p-value from the “protest” test. 16S, 16S rRNA gene amplicon sequencing; MTG, shotgun metagenomic sequencing; MTT, metatranscriptomic sequencing.


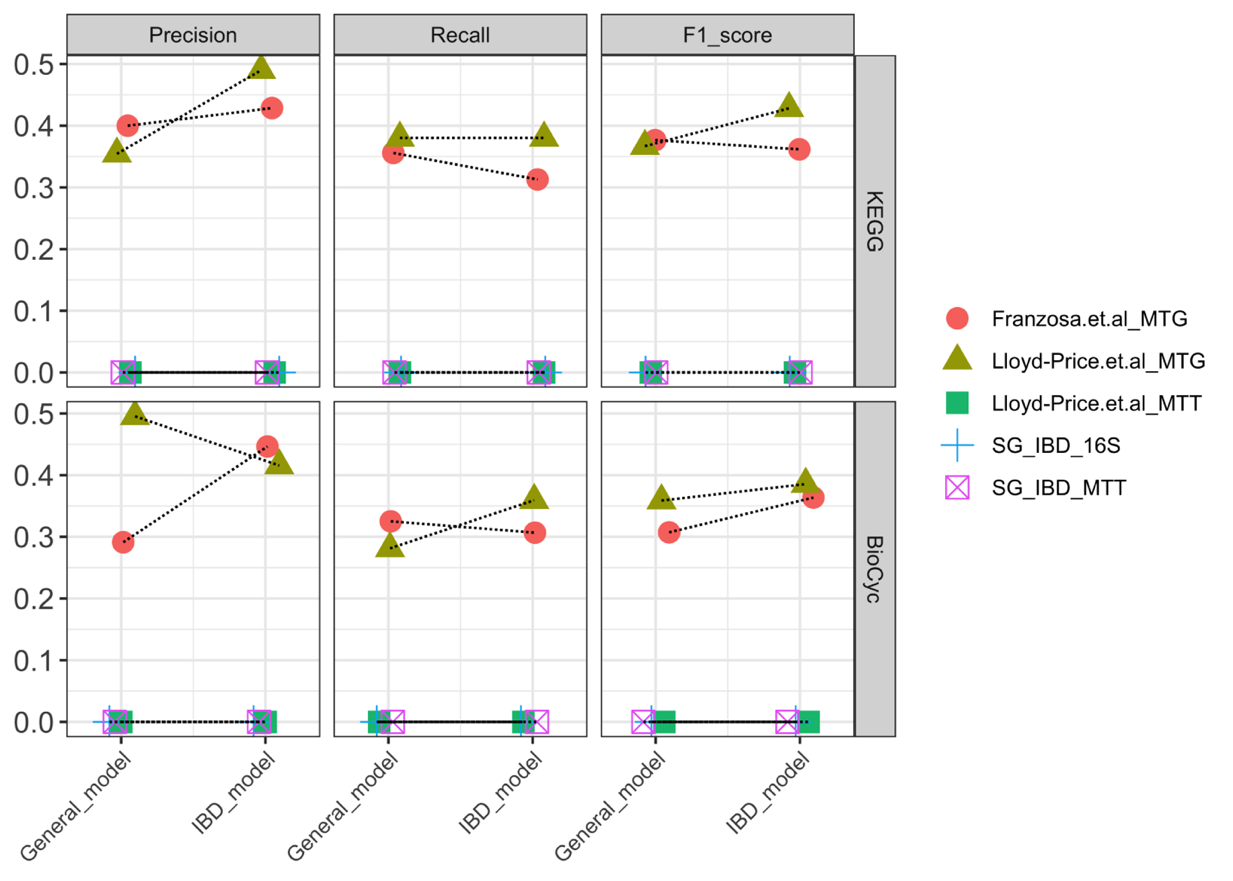


**Supplementary Figure S6**. Comparative analysis of precision, recall, and F1 score upon identifying differential metabolites with MelonnPan models constructed from either all datasets (general model) or IBD specific datasets (IBD_model). 16S, 16S rRNA gene amplicon sequencing; MTG, shotgun metagenomic sequencing; MTT, metatranscriptomic sequencing.
